# Supplementary material for: A new species of Oligodon Fitzinger, 1826 from the Langbian Plateau, southern Vietnam, with additional information on Oligodon annamensis Leviton, 1953 (Squamata: Colubridae)
Source: PeerJ. 2020 Jan 6;8:e8332. doi: 10.7717/peerj.8332 (PMC6951295; doi:10.7717/peerj.8332)
Supplement: Supplemental Information 1 [file peerj-08-8332-s001.docx]

**(I) 12S rRNA – 16S rRNA sequences of *Oligodon***

>ZMMUR14304rRNA [organism=Oligodon annamensis]

TACACCGCCCGTCCACCCTGCCACTAAAGTAAACACATTCATAATAACCCACATCAATAC

AAATCAGGGCAAGTCGTAACATGGTAAGCGTACTGGAAAGTGCGCTTAGAAACAAAAAGT

AGCTTACAAAAAGCATTCGGCCTACAACTGAAAGACATTACATCAATCTTTTTGAGCCAA

CAATAACGCACAAACATACACCAATAAAATAAACAAAGCATTTGACTAACTTAGTAGATG

AGATCGAACAGTAAACATAATCAGTACCGCAAGGGAAACAAACAAGCAATAAACAGCAAA

GACTAACCCTTGTACCTTTCGCATCATGGTCTAGCAAGAAATTAAAGACAAGAAGAATCA

TAGCCTTCACCCCGAAACCAGATGAGCTACTTTCAAGCAGCCAAAAGGGCTCACCCTTCT

CTGTAGCAAAAGAGTGGGAAGACTTAAAAGTAGAAGTGAAACGCCTATCGAATCTGGAGA

TAGCTGGCTACCCAAAAAAGAATATAAGTTCAACCACAGAACCAACAAGAACACGTTCTT

TCCTGTGGAAAATCAATAGGGGTCCAGCCCTATTGAAACAGGATACAACCTGAATTTGAG

AGAATAACTTAAACCTCGACCAGTAGACCTTAAAGCAGCCACCTAAAAAAATATCGTTAA

AGAATTTCCAAACTAATCCCAACACCAATTTCAAACTCCAAACCAACTAAAGGTAGACCC

ATAACAATGAGTACTATTATGCTAGAACTAATAATAAGACAACCTCTCTTCATGCACCTT

TCCACTAAACCGGACCAACCATTAGCCATTAACAGACCACAACAGGCATTAAACCAAACA

TTACACAACTTAAATCATACTGTTACCCCAACACAGGCGCATCAAAAAGAAAGATTTACA

ATTATAAAAGGAACTCGGCAAACAAAGATTCCAACTGTTTAACAAAAACATAACCTTTAG

ACCAACCAATATTAAAGGCAACGCCTGCCCAGTGAACATTAAACGGCCGCGGTACCCTAA

CCGTGCAAAGGTAGCATAATCATTTGTCTACTAATTATAGACCTGTATGAAAGGCAAAAT

GAGAATCTAGCTGTCTCTTATAATCAATCAATTAAACTGATCTCCTAGTAAAAAAGCTAG

AATTAAACCATAAGACCAGAAGACCCTGTGAAGCTTTAACTAAACTATTAAACCATATAA

TACCTACTTTCGGTTGGGGCGACCTTGGAAACAAAAAGAACTTCCAACAATGCAACCTAC

CTCGCACTACACCTAGGCCCACAAGCCAATTAATGACCCAGCAAAGCTGATAATTGAACC

AAGTTACTCCAGGGATAACAGCGCAATCCTCTTCAAGAGCCCATATCAAAAAGAGGGTTT

ACGACCTCGATGTTGGATCAGGACATCCTAATGGTGCAGACGCTATTAAGGGTTCGTTTG

TTCAACGATCAATAGTCCTACGTGATCTGAGTTA

>CBC01899rRNA [organism=Oligodon annamensis]

GATACCCCACTATTAACCCTACCCACCATAGCCAACCAGTCTATATACCGCCGTCGCCAG

CCCACCTCCTGAGAGCATAAAAGTGAGCCAAACAGTTCAACACTAACACGACAGGTCAAG

GTGTAACTAATGGGTGGGAACAAGATGGGCTACATTTTCTATTCAGAACAAACGAATAAA

CTTTGAAAAAATAACTGAAGGCGGATTTAGCAGTATATTAAGGACAAAACACTTAATCGA

AACCACCGCAATGGGGTGCGCACACACCGCCCGTCATCCCTGCCACTAAACTAACACATT

CATAATAACCTCATTAATACAAATCAGGGCAAGTCGTAACATGGTAAGCGTACTGGAAAG

TGCGCTTAGAAACAAAAAGTAGCTTACAAAAAGCATTCGGCCTACAACCGAAAGACATTA

CATCAATCTTTTTGAGCCAACAATAACGCACAAACATATACCAATAAAATAAACAAAGCA

TTTGACCAACTTAGTAGATGAGATCGAACAGTAAACATAATCAGTACCGTAAGGGAAACA

AACAAGCAATAAACAGCAAAGACTAACCCTTGTACCTTTCGCATCATGGTCTAGCAAGAA

ATTAAAGACAAGAAGAATCATAGCCTTCACCCCGAAACCAGATGAGCTACTTTCAAGCAG

CCAAAAGGGCTCACCCTTCTCTGTAGCAAAAGAGTGGGAAGACTTAAAAGTAGAAGTGAA

ACGCCTATCGAATCTGGAGATAGCTGGCTACCCAAAAAAGAATATAAGTTCAACCACAGA

ACCAACAAGAACACGTTCTTTCCTGTGGAAAATCAATAGGGGTCCAGCCCTATTGAAACA

GGATACAACCTGAATTTGAGAGAATAACTTAAACCTCAACCAGTAGACCTTAAAGCAGCC

ACCTAAAAAAATATCGTTAAAGAATTTCCAAACTAATACCAACACCAATTTCAAACTCCA

AACCAACTAAAGGTAGACCCATAACAATGGGTACTATTATGCTAGAACTAATAATAAGAC

AACCTCTCTTCATGCACCTTTCCACTAAACCGGACCAACCATTAGCCATTAACAGACCAC

AACAGGCATTAAACCAAACATTACACAACTTAAATCATACTGTTACCCCAACACAGGCGC

ATCAAAAAGAAAGATTTACAATTATAAAAGGAACTCGGCAAACAAAGATTCCAACTGTTT

AACAAAAACATAACCTTTAGATCAACCAATATTAAAGGCAACGCCTGCCCAGTGAACATT

AAACGGCCGCGGTACCCTAACCGTGCAAAGGTAGCATAATCACTTGTCTACTAATTATAG

ACCTGTATGAAAGGCAAAATGAGAATCTAGCTGTCTCTTATAATCAACCAATTAAACTGA

TCTCCTAGTAAAAAAGCTAGAATTAAACCATAAGACCAGAAGACCCTGTGAAGCTTTAAC

TAAACTATTAAACCATATAATACCTACTTTCGGTTGGGGCGACCTTGGAAACAAAAAGAA

CTTCCAACAATGCAACCTACCCCGCACTACACCTAGGCCTACAAGCCAATTAATGACCCA

GCAAAGCTGATAATTGAACCAAGTTACTCCAGGGATAACAGCGCAATCCTCTTCAAGAGC

CCATATCAAAAAGAGGGTTTACGACCTCGATGTTGGATCAGGACATCCTAATGGTGCAGA

CGCTATTAAGGG

>ZMMUR13364rRNA [organism=Oligodon lacroixi]

TTAACAAGACAATCAAATCACCCATTGTTCGCCAAATAACTACGAGTTACATCTTAAAAT

TAAAAGACTTGACGGTACCCCACAACAACCTAGAGGAGCCTGTCTAATAAATGATACTCC

ACGATTAACCCTACCCACCATAGCCCACCAGTCTATATACCGCCGTCGCCAGCCCACCTC

ATGAGAGAATAATAGTGAGCCAAATAGTTCCCACTAATACGACAGGTCAAGGTGTAACTA

ATGGGTGGGACCAAGATGGGCTACATTTTCTAACCCAGAACATACGAATAAACTCTGAAA

AAGAAACTGAAGGCGGATTTAGCAGTATATTAAGAACAAAATACTTAATTGAAGCCAACG

CAATGGGGTGCGCACACACCGCCCGTCATCCCTGCCACTAGACAAACACATCCATAATAA

CACACTAATACAAACCAGGGCAAGTCGTAACATGGTAAGCGTACTGGAAAGTGCGCTTAG

AAACAAAAAGTAGCTTACCAAAAGCATTCGGCCTACAACCGAACGACATTACACTAATCT

TTTTGAGCCGAAATCTAACGCACAAACATATACCAATAAACAAACAAAACATTTGACCAT

CCTAGTAGATGAGATCGAACAGCAAACCAACTTAGTACCGCAAGGGAAACTAAACAAGCA

ACAAACAGCAAAGATTAACCCTTGTACCTTTTGCATCATGGTCTAGCAAGAAACCAAAGA

CAAGAAGAATCAAAGCCTCCACCCCGAAACCAGATGAGCTACTTTAAAGCAGCCTAACGG

GCACACCCTTCTCTGTAGCAAAAGAGTGGGAAGACTTAAAAGTAGAAGTGAAACGCCTAC

CGAATCTGGAGATAGCTGGCTACCCAAAAAAGAATATAAGTTCTACCACAGAACTAACAA

GAATCTATTCTAACCTGTGGATAATCAATAGAGGTACAGCTCTATTGAAACAGGATACAA

CCTGAATTTGAAAGGAAAATTACACCTGCCCCCAGTAGGCCCTAAAGCAGCCACCTAACA

AAATATCGTTAAAGAATTATACAACTCTAAACCCAACACTAATCAAAAACTCCAAATCAA

CTAAAGGTAAGCCCATTATAATGGGTCCTACCATGCTAGAACTAATAATAAGACAATATC

TCTTCATGCACCTTTCTACTAAAACCGGACCAACCGCTAGCCATTAACAGACCACATTAG

GAATAAACTAAGCACTACACAACTTTAAACAAACTGTTACCCCAACACAGGCGCATCAAA

AAGAAAGATTAACCATTATAAAAGGAACTCGGCAAACAAAGACTTCAACTGTTTAACAAA

AACATAACCTTTAGACTAACCAATATTAAAGGCAACGCCTGCCCAGTGAACAATTAAACG

GCCGCGGTACCCTAACCGTGCAAAGGTAGCATAATCATTTGTCTACTAATTATAGACCTG

TATGAAAGGCAAAATGAAAGTCTAACTGTCTCTTATAATAAATCAATTAAACTGATCTCC

TAGTAAAAAAGCTGGAATTTAACCATAAGACCAGAAGACCCTGTGAAGCTTAAACTAAAC

TATTAAACCTCGTAATAATTACTTTCGGTTGGGGCGACCTTGGAAAAAAAGAGAACTTCC

AACAATACGACCTACCTCGTACTACCCACACAGGCCAACAAGCCAATTAATGACCCAGCA

AAGCTGATAATTGAACCAAGTTACTCCAGGGATAACAGCGCAATCCTCTTCAAGAGCCCA

TATCAAAAAGAGGGTTTACGACCTCGATGTTGGATCAGGACATCCTAATGGTGCAAACGC

TATTAAGGGTTCGTTTGTTCAACGATCAACAGTCCTACGTGATCTGAGTTA

>SIEZC20201rRNA [organism=Oligodon rostralis sp.nov.]

GGGCAAGTCGTAACATGGTAAGCGTACTGGAAAGTGCGCTTAGAAACAAAAAGTAGCTTA

CAAAAAGCATTCGACCTACAACCGAAAGACATTAATCAATCTTTTTGAGCCGACAATAAC

GCACAAACATACACAATATAACAAACAAAGCATTTGACCAACCTAGTAGATGAGATCGAA

CAGTAGACACAACCAGTACCGTAAGGGAAATAAACAAGCAATAAACAGCAAAGACTAACC

CTTGTACCTTTCGCATCATGGTCTAGCAAGAAACTAAAGACAAGAAGAATCATAGCCTAC

ACCCCGAAACCAGATGAGCTACTTTAAAGCAGCCAAAAGAGCCAACCCTTCTCTGTAGCA

AAAGAGTGGGAAGACTTAAAAGTAGAAGTGAAACGCCTATCGAATCTGGAGATAGCTGGC

TACCCAAAAAAGAATATAAGTTCAACCACAGAACCTACAAGAACACATTCTTTCCTGTGG

AAAATCAATAGGGGTTCAGCCCTATTGAAACAGGATACAACCTGAATTTGAGAGAACAAC

TAACCAAAAACCAGTAGACCTTAAAGCAGCCACCTAAAAAAATATCGTTAAAGAATTTTC

AAACTAATCCCAACACCAATTCCAAACTCCAAACCAACTAAAGGTAGACCCATAACAATG

GGTACTATTATGCTAGAACTAATAATAAGACAACCTCTCTTCATGCACCTTTCCACTAAA

CCGGACCAACCACTAGTCATTAACAGACCACACTAGGCATTCAACCAAACAATACACAAC

TTAAAATCATACTGTTACCCCAACACAGGCGCATCAAAAAGAAAGATTTACAATTATAAA

AGGAACTCGGCAAACAAAGATTCCAACTGTTTAACAAAAACATAACCTTTAGACCAACCA

ATATTAAAGGCAACGCCTGCCCAGTGAACATTAAACGGCCGCGGTACCCTAACCGTGCAA

AGGTAGCATAATCATTTGTCTACTAATTATAGACCTGTATGAAAGGCAAAATGAGAATCT

AACTGTCTCTTATAATTAACCAATTAAACTGATCTCCTAGTAAAAAAGCTAGAATTAAAC

CATAAGACCAGAAGACCCTGTGAAGCTTTAACTAAACTATTAAACCATGTAATACCTACT

TTCGGTTGGGGCGACCTTGGAAATAAAAAGAACTTCCAATAACACGACCTATCTCACACT

TCACCTTAGGCCTACAAGCCAATCAATGACCCAGCAAAGCTGATAATTGAACCAAGTTAC

TCCAGGGATAACAGCGCAATCCTCTTCAAGAGCCCATATCAAAAGGAGGGTTTACGACCT

CGATGTTGGATCAGGACATCCTAATGGTGCAGATGCTATTAAGGGTTCGTTTGTTCAACG

ATCAATAGTCCTACGTGATCTGAGTTC

**(II) cyt *b* sequences of *Oligodon***

>ZMMUR14304 [organism=Oligodon annamensis]

CCAACACATATTACTCGCATTTAACCTACTCCCAGTAGGATCCAATATCTCAACCTGATG

AAACTTCGGATCCATACTACTAGCCTGCCTAGCCCTACAATTTACAACTGGATTTTTCCT

AGCCATTCACTATACAGCCAATATCAACCTGGCCTTTTCATCCATCATCCACATCATACG

CGACGTCCCACACGGGTGAATCCTACAAAATCTTCACGCCATCGGCGCATCCATATTTTT

CATCTGTATCTACACCCACATCGCACGAGGACTTTACTACGGATCCTACATAAATAAAAA

CGTATGATTATCAGGAACCTCCCTCCTAATTATTCTAATAGCAACAGCCTTTTTCGGATA

CGTCCTTCCCTGAGGACAAATATCATTCTGAGCAGCCACAGTAATTACAAACCTATTAAC

CGCCGTACCCTACTTCGGCCTGACACTAACCACATGACTTTGAGGCGGATTTTCAATCAA

CGACCCAACATTGACCCGATTCTTCGCCCTTCACTTCATCCTACCATTCACCATTATCTC

CCTATCATCTATCCACATCATACTCCTCCACACAGAAGGTTCTAGCAACCCCCTAGGAAC

AAACTCGGACATTGATAAAATCCCATTCCATCCATATCACTCCCATAAAGATATACTAAT

AATAACCATTATCATAACATTATTATTCACCATTATATCGTTCACCCCAAATATCTTTAA

TGACCCAGAAAACTTCTCAAAAGCCAATCCCATAGTAACTCCACAACACATTAAACCAGA

ATGGTACTTCCTATTCGCTTACGGAATCCTACGATCCATCCCAAACAAATTAGGGGGGAC

AGTAGCCCTAATACTGTCCGTAATTATCCTAACCACAGCCCCATTCACCCACACATCACA

CCTACGACCCATAACCTTCCGACCCATTATACAATTTATATTCTGAACTATAATCGCCAC

ATTTATCACAATCACATGAGCAGCAACTAAACCAGTAGAACCCCCATTCACACTTATCGG

CCAAGCAACATCTATCCTATACTTCCTATTCTTCATCACAAACCCAATAATGGGCTGACT

AGAAAATAAAA

>CBC01899 [organism=Oligodon annamensis]

CCAACACATATTACTCGCATTTAACCTACTTCCAGTAGGATCCAATATCTCAACCTGATG

AAACTTCGGATCCATACTACTAGCCTGCCTAGCCCTACAATTTACAACTGGGTTTTTCCT

AGCCATTCACTATACAGCCAATACCAACCTGGCCTTTTCATCCATCATCCATATCATACG

CGACGTCCCACACGGATGAATCATACAAAATCTTCACGCTATCGGCGCATCCATATTTTT

CATCTGTATCTACACCCACATCGCACGAGGACTTTACTACGGATCCTACATAAATAAAAA

CGTATGATTATCAGGAACCTCCCTCCTAATTATTCTAATAGCAACAGCCTTTTTCGGATA

CGTCCTTCCCTGAGGACAAATATCATTCTGAGCAGCCACAGTAATTACAAACCTATTAAC

CGCCGTACCCCACTTCGGCCTGACACTAACCACATGACTTTGAGGCGGATTTTCAATCAA

CGACCCAACATTGACCCGATTCTTCGCCCTTCACTTCATCCTACCATTCACCATTATCTC

CTTATCATCTATCCACATCATACTTCTCCACACAGAAGGCTCTAGCAACCCCCTAGGAAC

AAACTCGGACATTGATAAAATTCCATTCCATCCATATCACTCCCATAAAGATATACTAAT

AATAACCATTATAATAACATTATTATTCACCATTATATCGTTCACCCCAAATATCTTTAA

TGACCCAGAAAACTTCTCAAAAGCCAATCCCATAGTAACTCCACAACACATTAAACCAGA

ATGGTACTTCCTATTCGCTTACGGAATCCTACGATCCATCCCAAACAAATTGGGGGGGAC

AGTAGCCCTAATACTGTCCGTAATTATCCTAACCACAGCCCCATTTACCCACACATCACA

CCTACGACCCATAACCTTCCGACCCATTATACAATTTATATTCTGAACTATAATCGCCAC

ATTTATCACAATCACATGAGCAGCAACCAAACCAGTAGAACCCCCATTCACACTTATCGG

CCAAGCAACATCTATCCTATACTTCCTATTCTTCATCACAAACCCAATAATGGGCTGACT

AGAAAATAAAA

>ZMMUR13364 [organism=Oligodon lacroixi]

CCAACACGCATTACTCCTATTCAACCTCCTCCCAGTAGGAACCAACATCTCAACCTGATG

AAACTTTGGGTCTATACTACTAGCTTGCTCAGCCCTACAAATTACAACCGGATTCTTCCT

GGCTATTCACTATACAGCTAACATTAACTTAGCCTTCTCATCTATCATTCACATCACACG

AGATGTCCCACACGGATGAATCATACAAAATCTTCACGCCATTGGCGCATCCATATTTTT

CATCTGCATCTACATCCACATTGCACGCGGGTTGTACTACGGATCCTACATAAACAAAAA

CGTATGATTATCAGGGACCTCTCTCCTAATCATCCTAATAGCAACAGCCTTCTTCGGATA

TGTCCTCCCTTGAGGACAAATATCATTCTGGGCCGCCACAGTAATTACAAACCTTCTAAC

AGCCGTACCCTACCTAGGCCTAACATTAACCACATGGCTCTGGGGCGGATTCTCAATCAA

TGACCCTACACTAACCCGATTCTTCGCCCTACACTTCATCCTACCATTCGCCATCATTTC

CATGACATCAATCCACATCATACTTCTCCACACAGAAGGCTCTAGCAACCCCCTAGGAAC

AAACTCGGACATTGACAAAATCCCATTCCACCCCTATCACTCCCACAAAGACATACTAAT

AATAACCATCATAATCACACTACTATTCACCATTATATCGTTTATCCCCAACATCTTTAA

TGACCCAGAAAACTTCTCAAAAGCTAACCCCCTAGTAACACCACAGCACATCAAACCAGA

ATGGTACTTCTTATTCGCATATGGCATCCTCCGATCCATCCCAAATAAACTAGGAGGAAC

CGTAGCCCTGGTACTATCCGTAACTATCCTAATCACAACACCATACACCCACACATCACA

CCTACGACCCATAACCTTTCGACCCCTCATACAAACAATATTCTGAACCCTGATCGCCAC

ATTCATCACAATCACATGAGCAGCCACTAAACCAGTAGAACCCCCATTCACACTAATTGG

CCAAACAACCTCCATCCTTTTCTTCTCATTCTTCATCATAAACCCATTGGTGGGGCACCT

AGAAAACAAAA

>SIEZC20201 [organism= Oligodon rostralis sp.nov.]

CCAACGCATATTACTTATATTTAACCTACTCCCAGTAGGATCCAATATCTCAACCTGATG

AAACTTCGGATCCATGCTACTAGCCTGCTCAGCCCTACAAATTACAACCGGGTTCTTCCT

AGCTATTCACTATACAGCCAACACTAACCTAGCCTTTTCATCCATTATCCACATCATACG

TGATGTCCCACATGGATGAATCATACAAAATCTACACGCCATCGGCGCATCCATGTTTTT

TATCTGCATCTACATCCATATCGCACGAGGACTTTACTACGGGTCCTACATAAATAAAAA

CGTATGATTATCGGGTACCTCCCTCCTAATTATCCTAATAGCAACAGCCTTTTTCGGATA

CGTTCTTCCCTGAGGACAAATATCATTCTGGGCAGCCACAGTAATTACAAACCTATTAAC

TGCCGTACCCTACTTCGGCCTAACACTAACCACATGACTTTGAGGCGGATTCTCAATCAA

CGACCCAACATTGACCCGATTCTTCGCTCTCCACTTTATCCTACCATTCACCATTATCTC

CTTATCATCAATCCACATCATACTTCTTCACACGGAAGGTTCTAGCAATCCTCTAGGAAC

AAACTCGGACATTGATAAAATCCCATTCCACCCGTATCACTCTTATAAAGATATACTAAT

AATGACTATCATCATAACATTATTATTCATCATTATATCGTTCACCCCAAACATCTTTAA

TGACCCAGAAAACTTCTCAAAAGCCAACCCCATGGTAACTCCACAACACATTAAACCAGA

GTGATACTTCCTATTCGCTTACGGAATCCTCCGATCCATCCCAAATAAACTAGGAGGAAC

AGTAGCCCTAATACTCTCCGTAATTATCCTAACTACAGCCCCATTCACCCACACATCACA

CCTACGACCCATAACCTTCCGACCCGTCATACAATTAATATTCTGAACCATAATCGCCAC

ATTTATCACAATCACATGAGCAGCAACCAAACCAGTAGAACCCCCATTCACACTTATCGG

CCAAACAACATCTGCCCTATACTTCCTATTCTTTATCACAAACCCTATACTGGGCTGACT

AGAAAATAAAA
